# Does social media promotion influence citation counts? A long-term follow-up to a randomised trial in a general neurosurgical journal

**DOI:** 10.1007/s10143-025-03904-4

**Published:** 2025-11-05

**Authors:** Albert Q. Schmidt, Sven Theiler, Mateo Tomas Fariña Nuñez, Victor Gabriel El-Hajj, Moira Vieli, Bianca Battilana, Alex Alamri, Katrin Rabiei, Laura Lippa, Claire Karekezi, Angelos Kolias, Carlo Serra, Luca Regli, Tiit Mathiesen, Victor E. Staartjes

**Affiliations:** 1 Department of Neurosurgery, Machine Intelligence in Clinical Neuroscience & Microsurgical Neuroanatomy (MICN) Laboratory, Clinical Neuroscience Center, University Hospital Zurich, University of Zurich, Frauenklinikstrasse 10, CH-8091 Zurich, Switzerland; 2 School of Medicine, Royal College of Surgeons in Ireland (RCSI), Dublin, Republic of Ireland; 3 Department of Spine Surgery, Schulthess Klinik, Zurich, Switzerland; 4 Department of Clinical Neuroscience, Karolinska Intitutet, Stockholm, Sweden; 5 Department of Neurosurgery, The Royal London Hospital, Barts Health NHS Trust, London, UK; 6 Department of Neurosurgery, Art Clinic, Uppsala, Sweden; 7 Department of Neurosurgery, Azienda Ospedaliero-Universitaria Senese, Siena, Italy; 8 Department of Neurosurgery, Rwanda Military Hospital, Kigali, Rwanda; 9 Division of Neurosurgery, Addenbrooke’s Hospital, University of Cambridge, Cambridge, UK; 10 University of Copenhagen, Copenhagen, Denmark; 11 Rigshospitalet, Copenhagen, Denmark

**Keywords:** Social Media, Twitter, X, Randomized Controlled Trial, Neurosurgery, Citations

## Abstract

Social media promotion has become mainstream for neurosurgical publications. Any effect of promotion on citation counts would significantly influence academia and is currently not well-studied. We previously reported that structured social media promotion of neurosurgical articles had no significant effect on citation counts, website visits, or PDF downloads at one- and two-years post promotion. In this study, we assess whether a longer follow-up period has altered these previously reported results, since citation counts typically follow a Poisson-like distribution, increasing gradually and often peaking several years after publication. We followed up the original 177 articles published in *Acta Neurochirurgica* between May and September 2020 which were randomised either to the social media intervention (single Twitter/X post, *n* = 89) or to the control group (no promotion, *n* = 88). The primary outcome (citation counts) and secondary outcomes (website visits and altmetrics) were reassessed 4.5 years post-promotion. Between-group comparisons were performed using Welch’s t-tests. A sensitivity analysis was conducted using negative binomial regression models, better approximating the non-linear distribution of citation counts. At 4.5 years, there was no significant difference between intervention and control groups for citation counts (12.76 ± 12.18 vs. 16.47 ± 21.92, *p* = 0.168) and website visits (1448 ± 1489 vs. 1503 ± 1692, *p* = 0.818). Altmetric scores were significantly higher in the intervention group (5.15 ± 3.88 vs. 1.74 ± 3.68, *p* < 0.001). The sensitivity analysis confirmed these findings, showing no significant difference in citation counts (IRR 0.78; 95% CI: 0.57–1.05; *p* = 0.101) but a significant increase in Altmetric scores (IRR 2.96; 95% CI: 2.09–4.19; *p* < 0.001). A longer observation time did not result in a significant difference in citation counts or website visits. Promoted articles were consistently associated with increased Altmetric scores. A low-intensity social media promotion may broaden visibility but does not influence long-term citations. More intensive promotion strategies have demonstrated short-term gains, but their ability to produce sustained long-term impact remains uncertain and warrants further investigation.

## Introduction

Social media has become a prominent tool in neurosurgery, with growing engagement from neurosurgical journals, departments and individual surgeons [6, 9, 12]. Despite limited evidence supporting social media’s effectiveness in promoting articles, nearly all major neurosurgical journals maintain an active presence on social media to promote articles for increasing viewership, downloads and citations. In an earlier publication, we observed that a structured social media campaign was associated with increased article website visits and PDF downloads compared to historical controls [13].

Since within neurosurgery there was little published evidence on the effects of social media promotion on citations, our group ran a randomised controlled trial (RCT) to investigate whether promoting neurosurgical publications through social media influenced their citation counts, website visits and PDF downloads after 2 years [17]. In it, we randomized 177 articles published in *Acta Neurochirurgica* from May through September 2020 into an intervention and control group. The intervention was promotion of the article through a single Twitter/X post. At one- and two-years post-promotion we found no significant impact of social media promotion on citation count, website visits or PDF downloads compared to the control.

Since citations accumulate slowly over time, a limitation of our original study was the relatively short follow-up period. Citation rates of articles often follow a Poisson distribution, with a delayed peak around years 4 to 5 [1, 3]. Our initial analysis assessed outcomes at 1- and 2-years post-publication, potentially missing delayed citations and long-term effects of social media promotion. Additionally, recent studies in neurosurgery have shown growing interest in alternative metrics (Altmetric scores) as potential indicators of research visibility [2]. In this follow-up, we therefore reassessed our RCT outcomes at 4.5 years post-promotion and additionally examined Altmetric scores to better characterise the long-term effects of social media promotion.

## Materials and methods

### Design

We performed a long-term 4.5-year follow-up of a RCT, the methods of which have been discussed in detail in our previous publication [17]. The original allocation was preserved – 177 non-invited articles published in *Acta Neurochirurgica* between May and September 2020 were randomised to a group with structured promotion though social media (intervention) or to a group left unpromoted (control). Randomisation was stratified by open access status, calendar quarters and the article type using permuted block randomisation.

The intervention consisted of a single post to the publishers then Twitter account (rebranded in 2023 to X). Each post featured the article title, relevant hashtags, a link to the article, a figure from the article and the flag of the article’s country of origin. Where available, the associated institutional or author accounts were tagged.

The primary endpoint of this study was the number of citations on Google Scholar at 4.5 years post-promotion.

Secondary endpoints were the number of website visits at 4.5 years post-promotion and Altmetric scores at 4.5-years post-promotion. Full-text PDF download data were not available for this analysis.

### Statistical analysis

All analyses followed the intention-to-treat principle. Continuous variables were reported as mean ± standard deviation and categorical variables as count (percentage). Welch’s t-tests compared the primary and secondary outcomes between the two study groups.

We also used an alternative statistical method to assess the influence of social media promotion on the citation counts. Citation and Altmetric counts generally follow a Poisson distribution or negative binomial distribution. So, to account for overdispersion, we conducted negative binomial regressions at 4.5 years as a sensitivity analysis. All models included group allocation (intervention vs. control) as the covariate. Results were reported as incidence rate ratios (IRRs) with 95% confidence intervals and corresponding p-values. Statistical analyses were performed using R Version 4.4.3 [11]. Two-tailed tests were regarded as statistically significant with *p* ≤ 0.05.

## Results

**Fig. 1:**
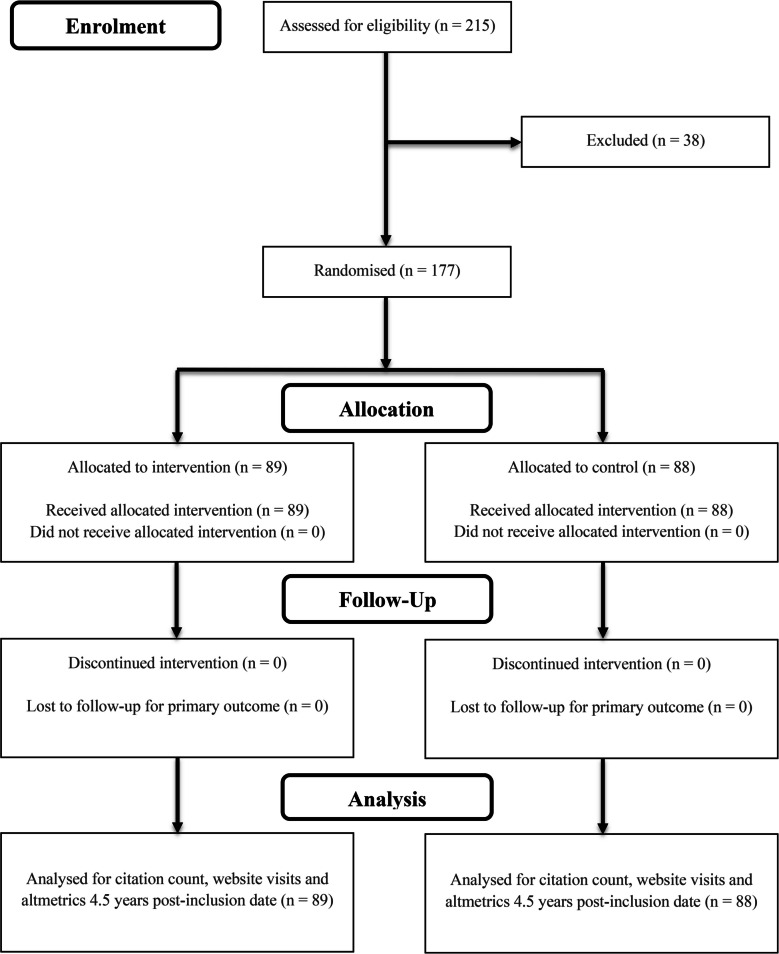
Flow diagram illustrating article allocation to either the intervention or control group. There was no loss to follow-up or discontinuation of the intervention after 4.5 years. Adapted from the CONSORT 2025 Statement flow diagram template [7]

**Fig. 2:**
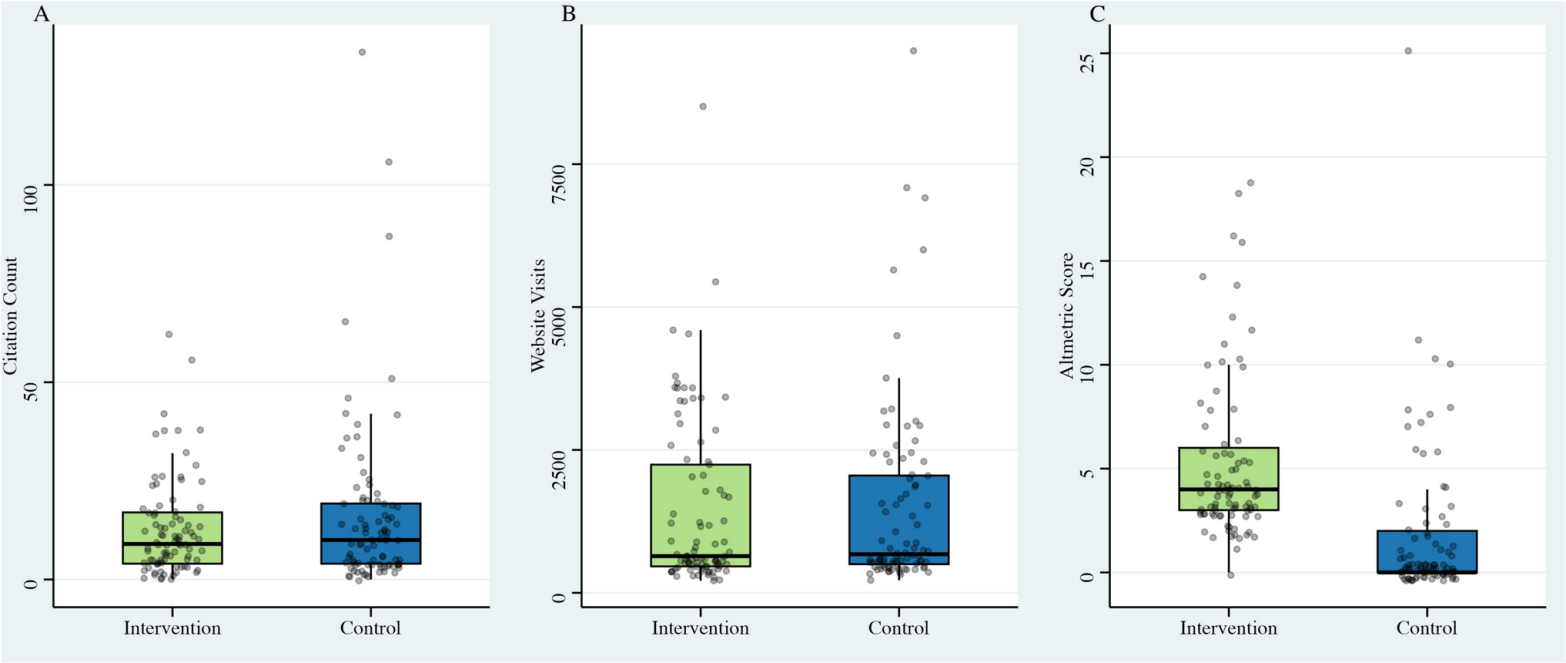
Boxplots illustrating the range of citations (**A**), website visits (**B**) and Altmetrics (**C**) for the intervention and control group at 4.5 years post-inclusion

The same cohort of 177 articles (Intervention: *n* = 89; Control: *n* = 88) from our original RCT publication were followed up 4.5 years after the final publication was randomized (Fig. 1). Baseline characteristics of the included articles can be found in Table 1. Full results are summarised in Table 2 and visualised in Fig. 2. Our previously published 1- and 2-year data are included for comparative purposes.

**Table 1:** Baseline characteristics of included articles

	Total	Intervention	Control
Number of Papers, *n* (%)	177 (100%)	89 (50.3%)	88 (49.7%)
Type of Article, *n* (%)			
Original Article	112 (63.3%)	57 (64.0%)	55 (62.5%)
Case Report	19 (10.7%)	9 (10.1%)	10 (11.4%)
How I do it	23 (13.0%)	12 (13.5%)	11 (12.5%)
Review Article	14 (7.9%)	7 (7.9%)	7 (8.0%)
Technical Note	9 (5.1%)	4 (4.5%)	5 (5.7%)
Editor's Choice, *n* (%)	1 (0.6%)	1 (1.1%)	0 (0%)
Open Access, *n* (%)	49 (27.7%)	25 (28.1%)	24 (27.2%)
Author in Tweet, *n* (%)	-	16 (18.0%)	-
Figure in Tweet, *n* (%)	-	85 (95.5%)	-

**Table 2:** Summary of outcomes at 1-, 2-, and 4.5-years following randomisation into a social media promotion (intervention) or no-promotion (control) group. Citation counts are based on Google Scholar data. Results are reported as means ± standard deviations. Data at 1 and 2 years were previously published [17]

	Intervention	Control	*p*
Citation Count			
1 year	1.85 ± 3.94	2.67 ± 6.65	0.322
2 years	5.35 ± 7.39	7.09 ± 12.10	0.249
4.5 years	12.76 ± 12.18	16.47 ± 21.92	0.168
Website Visits			
1 year	587 ± 568	591 ± 636	0.972
2 years	866 ± 856	896 ± 982	0.826
4.5 years	1448 ± 1489	1503 ± 1692	0.818
Altmetrics			
1 years	4.91 ± 3.99	1.62 ± 3.53	< 0.001
2 years	4.89 ± 3.96	1.73 ± 3.60	< 0.001
4.5 years	5.15 ± 3.88	1.74 ± 3.68	< 0.001

### Primary endpoint: citation count

At 4.5 years, the mean number of citations per article was 12.76 ± 12.18 in the intervention group and 16.47 ± 21.92 in the control group. However, this difference was not statistically significant (*p* = 0.168).

### Secondary endpoints: website visits and altmetrics

The mean number of website visits after 4.5 years was 1448 ± 1489 and 1503 ± 1692 in the intervention and control group, respectively. This difference was not statistically significant (*p* = 0.818).

Altmetric scores were significantly higher in the intervention group. At 4.5-years post-promotion, the mean altmetric score was 5.15 ± 3.88 in the intervention group compared to 1.74 ± 3.68 in the control group (*p* < 0.001).

### Sensitivity analysis

To better adjust for non-linear distribution of citation counts over time, negative binomial regression models were used as a sensitivity analysis and produced results consistent with the primary analysis shown in Table 3. Citation counts of articles in the intervention arm showed a lower, but statistically non-significant, incidence rate ratio (IRR 0.78; 95% CI: 0.57–1.05; *p* = 0.101). Altmetric scores were significantly higher in the intervention group, with an IRR of 2.96 (95% CI: 2.09–4.19; *p* < 0.001).

**Table 3:** Sensitivity analysis of citation and Altmetric outcomes at 1, 2 and 4.5 years. Incidence rate ratios (IRRs) were obtained from negative binomial regression models

	IRR	95% CI (low)	95% CI (high)	*p*
Citation Count				
1 year	0.69	0.49	0.99	0.044
2 years	0.75	0.55	1.04	0.082
4.5 years	0.78	0.57	1.05	0.101
Altmetrics				
1 year	3.02	2.13	4.29	< 0.001
2 years	2.83	2.00	4.01	< 0.001
4.5 years	2.96	2.09	4.19	< 0.001

## Discussion

Within the current literature, the long-term effects of social media on citation counts remain unclear. Most prior studies, including our own, assessed outcomes after just 1–2 years which is too brief to capture the true citation impact [3]. To address this, we extended the follow-up period of our RCT to 4.5 years to accurately assess long-term effects. Despite this extended observation period, we found no significant difference between promoted and control articles on citation counts. Previous randomised trials in other specialties have conflicting results, partly due to heterogeneity in the intensity of promotional strategies and differences in follow-up duration.

Fox et al. [4] and Widmer et al. [18] both evaluated article-level metrics at only one-month post-promotion. While Fox et al. [4] found no impact on views even after increasing the frequency of posts [5], Widmer et al. [18] employed a more intensive campaign across multiple platforms and observed significantly higher website visits and PDF downloads.

A similar high-intensity intervention was used by Luc et al. [10] by making a Twitter post than retweeting it by 11 delegates with a combined > 50,000 followers. They reported significantly increased citations and Altmetric scores after 1 year. However, it is unclear whether these effects were sustained over a longer follow-up period. Tonia et al. [15] made three posts per article at 0-, 2- and 12 weeks post-publication but found no impact on citations or download even after extending the follow-up period to 2 years [16]. Similar to our intervention, Ladeiras-Lopes et al. [8] promoted articles only through a one-off tweet. Unlike our results they found a significant association to higher citation rates over a 2.5-year follow-up period. However, their study pre-selected articles based on perceived relevance to the cardiovascular community and granted 24-h free access to promoted articles, possibly explaining the observed citation benefit and limiting attribution to the promotion alone.

Although we found no difference in citations or website visits, we did find a significant increase in Altmetric scores which is consistent with prior reports [8, 10]. A recent observational study of neurosurgical journals, Baig et al. [2] reported a statistically significant correlation between Altmetric scores, and citation count at three years. In our randomised study, although promoted articles consistently received higher Altmetric scores, this did not result into increased citation counts. Even after a sensitivity analysis using a negative binomial distribution – a distribution and modelling type that closely follows the distribution that citation counts over time exhibit, namely a overdispersed Poisson or negative binomial distribution – no differences between the randomized groups were found, providing relatively strong evidence that a one-time social media promotion likely does not change citation counts even on a long-term aspect.

This observation supports the idea that citations are mainly driven by the intrinsic scientific value of the published studies, regardless of attention that the study receives. Although this cannot be definitively proven within the scope of our trial, analyses have demonstrated a positive association between citation counts and expert-assessed research quality, suggesting that citations generally reflect scientific merit [14]. High-quality or highly relevant articles may naturally attract both attention and citations, whereas promoted articles—if lacking intrinsic value—may experience increased visibility but without a corresponding long-term increase in citations. This phenomenon is in our view a positive one, as artificially inflating citation counts through visibility alone would represent a pernicious distortion of academic impact. Our findings suggest that this potential risk of social media promotion does not materialise in the long term under a low-intensity promotion strategy.

## Limitations

The intervention was a low-intensity social media promotion, limited to a single post on one Twitter/X account. While this standardised approach helped minimise confounding, it does not reflect real-world practices, where sustained or multi-platform campaigns are more common and may have yielded different results. As discussed, such campaigns can increase short-term visibility and, in some cases, citations. Our findings should therefore be interpreted in the context of a deliberately low-intensity intervention, which may not be generalisable to more intensive approaches. Additionally, all articles were drawn from a single neurosurgical journal. These findings may not be generalisable to other journals or to different specialities. Additionally, the number of followers on the Twitter/X account used for promotion may influence the reach and potential impact of the intervention, and this can vary considerably between studies. At the time of this analysis, the *Acta Neurochirurgica* Twitter/X account had 4,524 followers (accessed May 28, 2025), which increased from 3,471 at the time of our initial analysis (On July 21, 2023).

## Conclusion

Our long-term follow-up of a randomised trial showed that promotion of articles through a single Twitter/X post did not lead to increased citation counts or website visits, even after 4.5 years. However, promoted articles received higher Altmetric scores, reflecting increased online visibility. The effect of social media on article performance is complex and likely depends on both the intensity of the promotion strategy and the duration of follow-up. We conclude that low-intensity social media promotion broadens reach but does not translate into increased citations in the long-term.

## Data Availability

The data in support of our findings can be obtained upon reasonable request from the corresponding author.
